# Prognostic Significance of miR-181b and miR-21 in Gastric Cancer Patients Treated with S-1/Oxaliplatin or Doxifluridine/Oxaliplatin

**DOI:** 10.1371/journal.pone.0023271

**Published:** 2011-08-18

**Authors:** Jingting Jiang, Xiao Zheng, Xiao Xu, Qi Zhou, Haijiao Yan, Xueguang Zhang, Binfeng Lu, Changping Wu, Jingfang Ju

**Affiliations:** 1 Department of Tumor Biological Treatment, The Third Affiliated Hospital of Soochow University, Changzhou, Jiangsu Province, People‘s Republic of China; 2 Department of Pathology, Stony Brook University, Stony Brook, New York, United States of America; 3 Key Laboratory of Stem Cell of Jiangsu Province, Key Laboratory of Clinical Immunology of Jiangsu Province, Soochow University, Suzhou, Jiangsu Province, People‘s Republic of China; 4 Department of Immunology, University of Pittsburgh, Pittsburgh, Pennsylvania, United States of America; Roswell Park Cancer Institute, United States of America

## Abstract

**Background:**

The goal of this study is to evaluate the effectiveness of S-1/Oxaliplatin vs. Doxifluridine/Oxaliplatin regimen and to identify miRNAs as potential prognostic biomarkers in gastric cancer patients. The expression of candidate miRNAs was quantified from fifty-five late stage gastric cancer FFPE specimens.

**Experimental Design:**

Gastric cancer patients with KPS>70 were recruited for the trial. The control group was treated with 400 mg/twice/day Doxifluridine plus *i.v.* with Oxaliplatin at 130 mg/m^2^/first day/4 week cycle. The testing group was treated with S-1 at 40 mg/twice/day/4 week cycle plus *i.v.* with Oxaliplatin at 130 mg/m^2^/first day/4 week cycle. Total RNAs were extracted from normal and gastric tumor specimens. The levels of miRNAs were quantified using real time qRT-PCR expression analysis.

**Results:**

The overall objective response rate (CR+PR) of patients treated with S-1/Oxaliplatin was 33.3% (CR+PR) *vs.* 17.6% (CR+PR) with Doxifluridine/Oxaliplatin for advanced stage gastric cancer patients. The average overall survival for patients treated with S-1/Oxaliplatin was 7.80 month vs. 7.30 month with patients treated with Doxifluridine/Oxaliplatin. The expression of miR-181b (*P* = 0.022) and miR-21 (*P* = 0.0029) was significantly overexpressed in gastric tumors compared to normal gastric tissues. Kaplan-Meier survival analysis revealed that low levels of miR-21 expression (Log rank test, hazard ratio: 0.17, CI = 0.06–0.45; *P* = 0.0004) and miR-181b (Log rank test, hazard ratio: 0.37, CI = 0.16–0.87; *P* = 0.018) are closely associated with better patient's overall survival for both S-1 and Doxifluridine based regimens.

**Conclusion:**

Patients treated with S-1/Oxaliplatin had a better response than those treated with Doxifluridine/Oxaliplatin. miR-21 and miR-181b hold great potential as prognostic biomarkers in late stage gastric cancer.

## Introduction

Gastric cancer remains one of the most common forms of cancer worldwide with approximately 870,000 new cases and 650,000 deaths per year. Gastric cancer is one of the most common causes of cancer death in China [1]. The early clinical detection of gastric cancer was less than 15%, and about 85% cases were advanced gastric cancer [2]. The average mortality rate of gastric cancer in China is over 26% for the male and 15% for the female. Gastric cancer is a rather complex and enigmatic disorder and many factors likely lead to the development of the disease. Epigenetics plays key role in gastric cancer development and mounting evidence showed that non-coding miRNAs are one of the major contributors to gastric cancer.

miRNAs are a class of small noncoding RNA of 20–22 nucleotides in length, which are processed from larger pre-miRNAs by the RNase III enzyme Dicer (DICER1) into miRNA duplexes [3]. One strand of this duplex associates with the RNA-induced silencing complex (RISC), whereas the other strand is generally degraded by cellular nucleases [3]. The miRNA–RISC complex binds to specific mRNA targets, leading to translational repression or cleavage of these mRNAs. Thus, miRNAs modulate protein expression by promoting RNA degradation, inhibiting mRNA translation, and in some cases, affecting transcription. Although miRNA-mediated mRNA degradation occurs in mammals, most mammalian miRNAs are thought to repress target gene expression at the translational level [4], [5], [6] via imperfect base-pairing to the 3′-untranslated regions (3′-UTRs) of their target mRNAs. This form of translational regulation provides the cell with a more precise, immediate and energy-efficient way of controlling the expression of a given protein [7] as it induces rapid changes in protein synthesis without excess transcriptional activation and subsequent steps in mRNA processing. Additionally, translational control of gene expression has the advantage of being readily reversible, providing the cell with great flexibility in responding to various stresses.

Fluoropyrimidine based chemotherapy (e.g. S-1/Oxaliplatin and Doxifluridine/Oxaliplatin) remains as a major treatment option for advanced gastric cancer patients. Previous studies have discovered a number of miRNAs associated with chemoresistance to 5-FU and S1 based therapy in colorectal cancer [8], [9], [10], [11], [12], [13]. S-1 is a novel oral dihydropyrimidine dehydrogenase (DPD) inhibitory fluoropyrimidine (DIF) based on a biochemical modulation of 5-fluorouracil (5-FU); S-1 contains tegafur (FF) and two types of enzyme inhibitor, 5-chloro-2,4-dihydroxypyridine (CDHP) and potassium oxonate (Oxo) in a molar ratio of 1∶0.4∶1. Doxifluridine is a fluoropyrimidine derivative and is activated to 5-fluorouracil by uridine phosphorylase, which is more highly expressed in malignant cells. A number of reports have demonstrated the importance of miRNAs in gastric cancer [14], [15], [16], [17]. However, currently there is no study on miRNAs related to flupyrimidine based chemotherapy treatment. There is an urgent need to discover prognostic biomarkers to assist the clinical management of advanced gastric cancer as this will help to select patients who will have survival benefit from the treatment, avoid the toxicity to non-responders, and reduce the healthcare cost for patients.

Although several miRNA profiling studies have been reported to reveal the importance of miRNA in gastric cancer with impact on cell cycle control, apoptosis, tumor invasion and metastasis [14], [17], currently there is no report to link miRNA with chemotherapeutic treatments with S-1/Oxaliplatin and Doxifluridine/Oxaliplatin. In this study, we systematically investigate the relationship of selected candidate miRNAs (e.g. miR-21, miR-181b, miR-192, miR-140, let-7g) in terms of their clinical utility in gastric cancer using archival gastric cancer FFPE specimens. These miRNAs have been previous reported to be associated with fluoropyrimidine based chemoresistance in colorectal cancer [9], [10], [11], [12], [13]. Pre-chemotherapy samples were chosen from the S-1/Oxaliplatin and Doxifluridine/Oxaliplatin in advanced gastric cancer. We have previous demonstrated that miRNAs are rather stable in FFPE samples and it is ideal for biomarker discovery [18]. Patients treated with S-1/Oxaliplatin had a better response than Doxifluridine/Oxaliplatin. However, there is no difference in overall patient's survival. We discovered that miR-21 and miR-181b are significantly associated with gastric cancer outcome with S-1/Oxaliplatin based chemotherapy. To our best knowledge, this is the first report to demonstrate the prognostic significance of miRNAs in gastric cancer related to S-1/Oxaliplatin and Doxifluridine/Oxaliplatin treatment. miR-21 and miR-181b hold great potential as prognostic biomarkers in late stage gastric cancer.

## Methods

### Patients and Samples

Clinical sample cohorts used for this study were approved by the Institution Review Board of Third Affiliated Hospital of Soochow University. Paraffin blocks containing formalin-fixed specimens of the tissues (FFPE) were acquired from the archival collections of the Department of Pathology, and used for subsequent RNA extraction. The characteristics of these patients are shown in Table 1.

**Table 1:** Clinical and pathologic parameters of gastric cancer patients treated with Doxifluridine/Oxaliplatin or S-1/Oxaliplatin.

Clinical/Pathologic Parameters	Doxifluridine/Oxaliplatin	S-1/Oxaliplatin	z/t-test	*P* value
**Sex**				
Male	16	23	1.139	0.254
Female	11	5	1.618	0.106
**Age**	Average: 63.3	Average: 62	1.027	0.31
**Diagnosis**				
No metastasis	4	7	0.940	0.347
Metastasis	23	21	0.301	0.763
**Tumor size** (cm^2^)	48.9	60.8	0.678	0.500
**Histology**				
Low differentiation	9	4	1.502	0.133
High differentiation	18	24	0.935	0.349
**Tumor stage**				
Stage III	3	3	0	1.000
Stage IV	24	25	0.143	0.886
**Treatment history**				
No	22	19	0.469	0.638
Yes	5	9	1.115	0.265
**KPS score**	82.0±7.1	80.7±6.8	0.66	0.514

### RNA Isolation

Using archival FFPE tissues, separate areas of solid tumor and normal gastric epithelium were identified using the corresponding Hematoxylin and Eosin stained sections and cores measuring 1.5 mm in diameter and 2 mm in length (approximately 0.005 g) were extracted. Subsequently, the samples were treated with deparaffinization, hydration, proteinase K, and ultimately total RNAs were isolated by using the TRIZOL reagent ( Invitrogen, CA, USA ) [18].

### Real time qRT-PCR analysis of miRNA expression

The miR-21, miR-140, let-7g, miR-181b, miR-200c, miR-192 specific primers and the internal control RNU44 gene were purchased from Applied Biosystems (CA, USA). cDNA synthesis was performed by the High Capacity cDNA Synthesis Kit (Applied Biosystems) with miRNA specific primers. Real time quantitative RT-PCR (qRT-PCR) was carried out on an Applied Biosystems 7500 Real time system (ABI 7500HT instrument) with miRNA specific primers by TaqMan Gene Expression Assay. Expression of miRNAs values was normalized according to the internal RNU44 control, and the relative expression values were plotted.

### Statistical Analysis

All statistical analysis was performed using GraphPad Prism software 5.0. Gene expression ΔCt values of miRNAs from each sample were calculated by normalizing according to internal control RNU44 expression, and relative quantification values were plotted. The differences between tumor and normal tissues were analyzed by Wilcoxon matched-pairs test. A Kaplan-Meier survival curve was generated by performing a Cox proportional hazards regression analysis to evaluate the expression level of miR-21 and miR-181b with survival rate. Statistical significance was set up to *P*<0.05 in each test.

## Results and Discussion

Tremendous amount of efforts have been focused on discovering predictive and prognostic biomarkers for gastric cancer [19]. Due to the disease complicity of gastric cancer, it still remains a major challenge to identify clinical useful biomarkers. With the recognition of the broad impact of miRNAs on multi targets and pathways, it is of our interest to identify a new class of predictive and prognostic biomarkers in gastric cancer based on miRNAs. There are several advantages of using miRNAs as biomarker compared to mRNA as miRNAs have broad regulatory function, relatively small numbers and better stability in archival FFPE samples [18]. We report here that expression levels of miR-181b and miR-21 constitute strong prognostic factors in advanced gastric cancer patients on both S-1/Oxaliplatin and Doxifluridine/Oxaliplatin regimens. The Clinical and pathologic parameters of gastric cancer patients treated with Doxifluridine/Oxaliplatin or S-1/Oxaliplatin were listed in **Table 1**.

### Differential expression of miRNAs in gastric cancer

**Figure 1:**
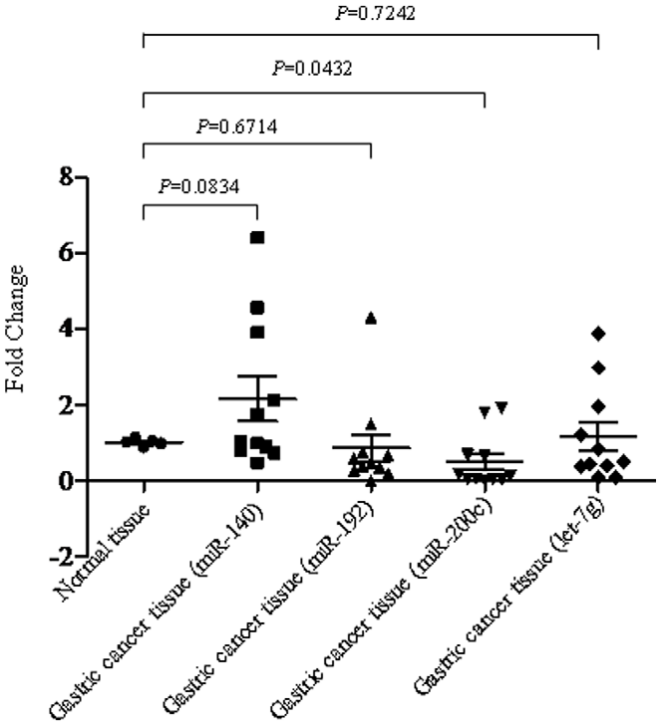
Expression levels of miR-140, miR-192, miR-200c, let-7g in both normal and gastric cancer specimens.

**Figure 2:**
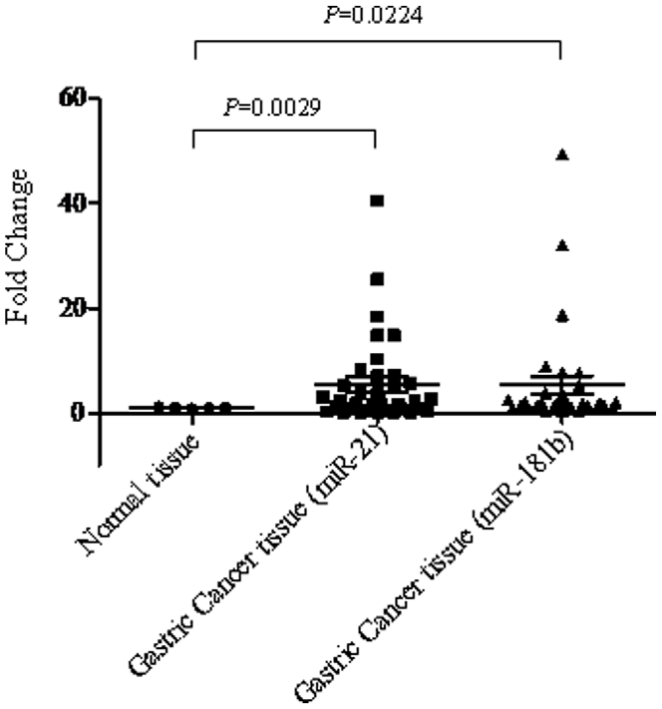
Expression levels of miR-21 and miR-181b in both normal and gastric cancer specimens.

We carefully selected a number of miRNA candidates that have been previous reported to be associated with fluoropyrimidine based chemoresistance in colorectal cancer [9], [10], [11], [12], [13]. The expression levels of miRNAs were quantified via real time qRT-PCR analysis using total RNA extracted from gastric cancer FFPE specimens and normal gastric tissues as controls. The miRNA expression levels were further normalized using internal control RNU44. Most of the miRNAs (e.g. miR-140, miR-200c) we have quantified shown differential expression between normal and tumor tissues (**Figure 1**). The expression of an important miRNA, miR-200c, was significantly reduced in gastric cancer. miR-200c has been reported to directly associated with cheomosensitivity and epithelial-to-mesenchymal transition (EMT) [20]. The expression of miR-181b (*P* = 0.022) and miR-21 (*P* = 0.0029) was most significantly overexpressed in gastric tumors compared to normal gastric tissues (**Figure 2**). Our results are consistent with previous reports that these miRNAs were deregulated in gastric cancer [21].

### Association of miR-21 and miR-181b with response to S-1/Oxaliplatin and Doxifluridine/Oxaliplatin treatment regimen

The overall objective response rate (CR+PR) of patients treated with S-1/Oxaliplatin was 33.3% (CR+PR) *vs.* 17.6% (CR+PR) with Doxifluridine/Oxaliplatin for stage III and IV gastric cancer patients. We have selected several miRNAs that have been shown to be associated with fluoropyrimidine based chemoresistance in colon cancer [9], [10], [11], [12], [13] and systematically evaluated their potential link with Doxifluridine/Oxaliplatin and S-1/Oxaliplatin in gastric cancer. The low expression of miR-21 (*P* = 0.001) and miR-181b (*P* = 0.001) were significantly associated with the S-1/Oxaliplatin responders (**Table 2**). This is highly consistent with previous report that miR-181b expression was associated with S1 treatment in colorectal cancer [8]. It has been reported that miR-181b was associated with multi drug resistance (MDR) by targeting BCL2 [22]. With regards to miR-21, our results were consistent with a recent report that suppression of miR-21 by anti-miR sensitizes pancreatic cancer cell lines to Gemcitabine (a fluoropyrimidine analog) treatment [23]. However, the favorable response did not impact patient's overall survival as the average overall survival for patients treated with S-1/Oxaliplatin (7.80 month) was similar with patients treated with Doxifluridine/Oxaliplatin (7.30 month). This is not surprising as often time favorable response does not translate to survival benefit.

**Table 2:** Association of miR-21 and miR-182b with response to S-1/Oxaliplatin treatment based on two-way ANOVA analysis.

miRNA	S-1/Oxaliplatin (Treatment cycle)	*P* Value
miR-21	Cycle 3	0.001
miR-181b	Cycle 3	0.017

### miR-21 and miR-181b were significantly associated with patient's survival

**Figure 3:**
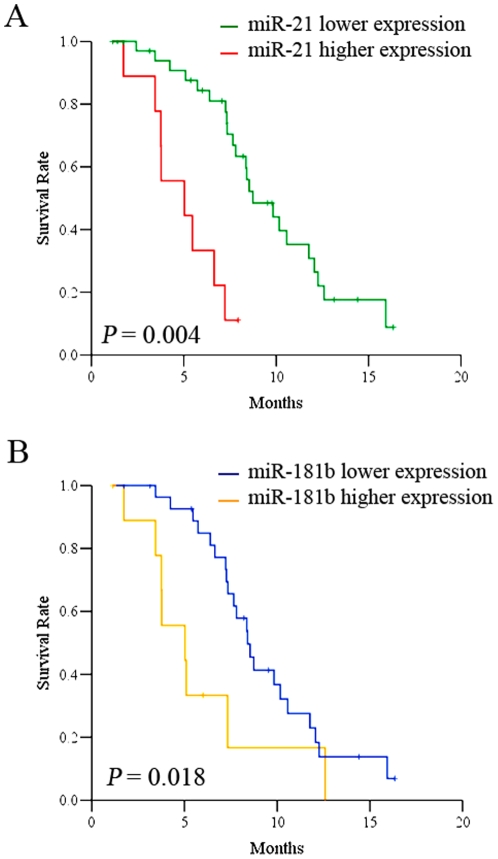
Kaplan-Meier overall survival curves of patients treated with S-1/Oxaliplatin and Doxifluridine/Oxaliplatin in association with miR-21 (A) and miR-181b (B).

To determine whether some of these miRNAs have potential as prognostic biomarker in gastric cancer, we performed patient's survival analysis using Kaplan-Meier survival curve by Multi variant Cox regression analysis. Kaplan-Meier survival analysis by Cox regression revealed that low levels of miR-21 expression (Log rank test, hazard ratio: 0.17, CI = 0.06–0.45; *P* = 0.0004) and miR-181b (Log rank test, hazard ratio: 0.37, CI = 0.16–0.87; *P* = 0.018) are closely associated with better patient's overall survival for both S-1 and Doxifluridine based regimens (**Figure 3**). The importance of miR-21 and miR-181b in gastric cancer was strongly supported by a recent report that the expressions of miR-21 and miR-181b were activated by STAT3 mediated by IL-6. miR-21 and miR-181b act as an epigenetic switch to inhibit PTEN and CYLD tumor suppressors, leading to increased NF-kB activity required to maintain the transformed state [24]. We also revealed that the expression of both miR-21 and miR-181b is positively associated in terms of expression value based on the statistical analysis (Pearson correlation r = 0.38, *P* = 0.018; Spearman correlation r_s_ = 0.38, *P* = 0.018). It is quite conceivable that miR-21 and miR-181b act as an epigenetic switch in gastric cancer development and contribute to chemoresistance by modulating key target tumor suppresser genes such as PDCD4, ANP32A and SMARCA4 genes [25]. Elevated miR-21 expression has been reported in many tumor types, suggesting the importance of miR-21 as a bona-fide oncogene. It has been reported the miR-21 was associated with *H. pylori* infection and gastric cancer development, suggesting that miR-21 could be the potential epigenetic link of inflammation of *H. pylori* infection and tumor initiation of gastric cancer [26].

In conclusion, we report here for the first time that the expression of miR-181b and miR-21 were associated with S-1/Oxaliplatin and Doxifluridine/Oxaliplatin treatment and patient survival. It further supports the notion for the potential importance of miR-181b and miR-21 in gastric cancer disease development. It establishes a foundation to further explore the underlining molecular mechanism of gastric cancer development involved with miR-181b and miR-21, and to fully validate our findings to the large cohort, multi-center clinical trial patients. miR-181b and miR-21 may also have potential to be as novel therapeutic targets in gastric cancer.
